# Small-Target Complex-Scene Detection Method Based on Information Interworking High-Resolution Network

**DOI:** 10.3390/s21155103

**Published:** 2021-07-28

**Authors:** Yongzhong Fu, Xiufeng Li, Zungang Hu

**Affiliations:** Engineering Institute of Advanced Manufacturing and Modern Equipment Technology, Jiangsu University, Zhenjiang 212013, China; lxf990309@163.com (X.L.); cadm@163.com (Z.H.)

**Keywords:** target detection, pose estimation, neural network, deep learning, visual inspection

## Abstract

The CNN (convolutional neural network)-based small target detection techniques for static complex scenes have been applied in many fields, but there are still certain technical challenges. This paper proposes a novel high-resolution small-target detection network named the IIHNet (information interworking high-resolution network) for complex scenes, which is based on information interworking processing technology. The proposed network not only can output a high-resolution presentation of a small target but can also keep the detection network simple and efficient. The key characteristic of the proposed network is that the target features are divided into three categories according to image resolution: high-resolution, medium-resolution, and low-resolution features. The basic features are extracted by convolution at the initial layer of the network. Then, convolution is carried out synchronously in the three resolution channels with information fusion in the horizontal and vertical directions of the network. At the same time, multiple utilizations and augmentations of feature information are carried out in the channel convolution direction. Experimental results show that the proposed network can achieve higher reasoning performance than the other compared networks without any compromise in terms of the detection effect.

## 1. Introduction

With the development of CNNs (convolutional neural networks), their application in the field of target detection has gradually increased, and new methods have been emerging one after another. The latest research includes the HRNet [1,2] based on pose estimation, cascade RCNN [3,4] developed on the basis of IoU (Intersection over Union), CenterNet [5,6], and CornerNet-Lite [7]. At present, since a high detection performance is required in real-world application scenarios, it is a common practice to use a method developed based on a faster RCNN [8] for performance improvement and optimization. For instance, the cascade RCNN mainly analyzes the influence of the IoU threshold selection on results at different stages; the HRNet network achieves a high-resolution presentation by using parallel multiresolution networks and multiscale information fusion on the basis of learning the underlying information exchange; the DenseNet network [9] provides a high-performance network by performing information fusion between channels and employs the ResNext [10] and Xception [11] models to use the convoluted data of every channel.

In the current applications in various scenarios, a test is first carried out on the above-mentioned general networks to find which network strategy suits the best for a specific task and dataset. Currently, there are multitudes of network strategies to choose from. The testing process can show which strategies are suitable for a particular task and which are not. Therefore, it is necessary to test multiple schemes before an effective strategy and a network type are selected. On this basis, a particular network is built according to the requirements of a specific scene and good detection performance can be obtained through repeated network optimization and enhancement. However, the research and development process is complex and time-consuming and it is hard to achieve a good balance between detection performance and reasoning performance.

Following the design concepts reported in the previous studies, this paper designs a novel network architecture named the IIHNet (information interworking high-resolution network) to overcome technical difficulties, such as a lack of rich information, complex environment, small target size, and occlusion, which affect the small-target detection performance in some scenarios. In some scenes, such as a construction site, the proposed network architecture can be used to identify and verify small targets, for instance, the human body or the human head. Experimental results show that the usage of the IIHNet strategy can provide excellent detection performance by effectively using the information of all channels and achieve significant improvement in reasoning performance while maintaining high-resolution presentation. Therefore, the proposed simple target detection network that is designed according to specific task requirements can achieve excellent performance in small-target detection in complex scenes.

## 2. Related Studies

In the field of deep learning-based target detection, detectors mostly adopt two architectures: one-stage architecture represented by SSD [12,13,14,15,16] and YOLO series [17,18,19] and two-stage architecture represented by the faster RCNN series [3,8,20] and its improved version. In the target-detection tasks with a high requirement for reasoning performance but a low requirement for detection performance, the one-stage detector architecture is preferred. In contrast, the two-stage detector architecture is preferred when there is a high requirement for detection performance, but the requirement for reasoning performance is not very high. However, when the requirements for both reasoning performance and detection performance are high, two-stage detector architecture yields better results than the one-stage detector architecture. Both have certain problems when it refers to the small target detection problem [21], where the one-stage approach is mainly due to its low detection accuracy and fixed sampling method, which makes it difficult to detect when objects are too close or too small to each other; similarly, the two-stage approach requires a fine global effective stride (GES) when detecting small targets, this leads to a very large memory overhead.

Currently, to solve the problem of small target detection based on deep learning, researchers have proposed more novel methods, which have achieved good performance on some typical datasets such as MS-COCO and PASCAL-VOC [22]. Guo et al. [23] proposed an effective and stable single-stage detector called CenterNet++, which consists of three modules. The feature refinement module is mainly used to extract multiscale contextual information; the feature pyramid fusion module is responsible for generating powerful semantic information; the head enhancement module can eliminate the impact of the complex background. Experiments show that the model achieves a state-of-the-art performance in the task of detecting the position of small target ships in SAR image datasets. In addition, this author also proposed a rotational Libra R-convolutional neural network (CNN) method for balancing the feature, sample, and objective level of a neural network, which achieves state-of-the-art accuracy on the DOTA dataset [24]. Bosquet et al. [25] proposed STDnet, a convolutional neural network focused on the detection of small objects that are defined as those under 16 pixels × 16 pixels, the authors proposes a novel early visual attention mechanism called the region context network (RCN), which processes only the most promising regions and discards the rest of the input image, allowing the STDnet to keep high resolution feature maps in deeper layers providing low memory consumption and higher frame rates.

In the IIHNet network used in this study, the data augmentation strategy was adopted at the stage of data preprocessing. After convolution was performed in the initial layer of the network, the convolution operations on the features of three different resolutions were performed simultaneously, and the information from the three convolution channels was further integrated to maintain the uninterrupted exchange of information in the horizontal and vertical directions of the network. Through repeated fusion and information processing, the underlying convolution features were utilized to the largest possible extent. The network was applied to the task of human detection of small targets in construction site scenes, experimental results show that the proposed network could reduce the reasoning time by 50% on average compared to the other classical networks without making any compromise in detection performance.

## 3. Proposed Model

This section introduces the IIHNet detection architecture and provides all related details. As there are multiple layers in a deep convolution network, the information presentation ability of each layer of the network depends on the richness of information obtained from the previous layer; therefore, the first principle of network pruning is channel reduction from back to front. Based on the MMDetection object detection toolbox, a network is designed and tested from the aspects of data augmentation and network structure. 

**Figure 1:**
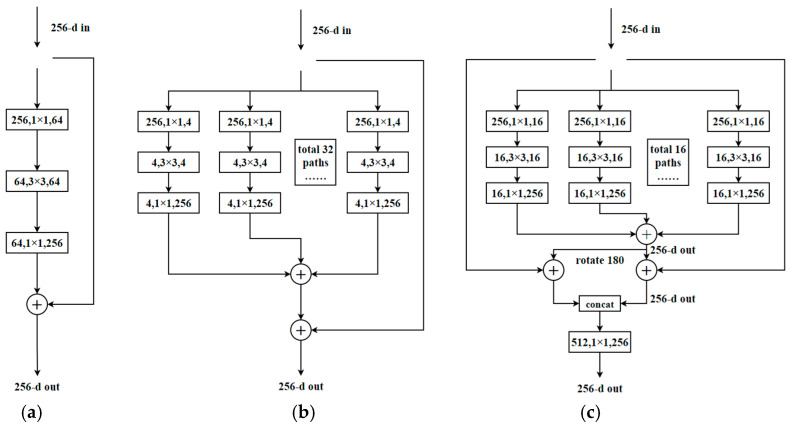
Evolution of convolution networks; (**a**) a block of ResNet; (**b**) a block of ResNeXt; (**c**) a block of ResNeXt-RC.

A network structure called the ResNeXt-RC that is based on ResNet [26] and ResNeXt networks was designed and it is shown in Figure 1a,b. The proposed network structure first regrouped channels of the previous two networks and used 16 × 16 convolution groups, then fused the information in two ways, using the concatenation and summing operations, and, finally, used a 1 × 1 convolution to reduce the channels number and fuse the information. The proposed ResNeXt-RC structure is presented in Figure 1c.

In the stage of feature information extraction and fusion, a widely used way of superimposing and combining information of channels was adopted to maximize the utilization of information. In order to use the convolution network to detect better the key parts of the human body or the human head, an efficient and simple IIHNet network was designed. Considering that the key features of detection targets in the dataset used in this study were sensitive to color information and that the main targets were relatively small, branches with an excessively low resolution were not needed. So, the network was divided into three resolution channels (from high-to-low resolution) for synchronous convolution. The convolution layer of the network is denoted as *N_ij_*, where *i* (*i* = 1, 2, 3) represents the resolution (resolution 2 is a half of resolution 1, and resolution 3 is a half of resolution 2) and *j* (*j* = 1, 2, 3) represents the information fusion stage. Thus, the convolution processes of the three convolution channels are as follows:

*N*_10_ → *N*_11_ → *N*_12_ → *N*_13_ (high-resolution)
   → *N*_21_ → *N*_22_ → *N*_23_ (sub-resolution)
    → *N*_31_ → *N*_32_ → *N*_33_ (low-resolution) (1)

The network structure consists of 10 convolution layers, where *N*_10_ is the initial convolution module of the network. After the convolution operation in the initial convolution module was finished, the next convolution operation was performed in the three convolution channels. Further, *N*_11_, *N*_12_, and *N*_13_ represent the convolution modules of the first, second, and third information interworking stages in the high-resolution channel, respectively; *N*_11_, *N*_21_, and *N*_31_ represent the convolution modules of the first information interworking stage in the high-resolution, subresolution, and low-resolution channels, respectively. The meanings of other annotations can be inferred in the same way. 

**Figure 2:**
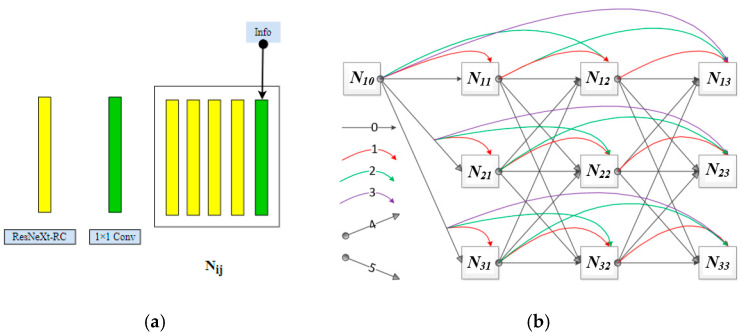
(**a**) Design of the network convolution module *N_ij_*; (**b**) multiscale parallel information exchange network structure, the IIHNet.

In the IIHNet network structure, information exchanges mainly occur in two directions, the horizontal direction (representing resolution channel) and the vertical direction (information interworking), as shown in Figure 2a. Each *N_ij_* module consists of four ResNeXt-RC structures and one 1 × 1 convolution block, which is used to perform information interworking for channel reduction. In Figure 2a, “*Info*” represent fused information, which includes the same resolution convolution information in the horizontal direction and the convolution information with different resolution at the same stage in the vertical direction, we adopted concatenated operation to generate high dimension features with these multiscale information to learn the relationship of channels, so that it improves the representational power. As shown in Figure 2b, the curves have different meanings, for instance, curves “0,” “1,” “2,” and “3” represent different transmission distances of information within the same convolution channel, which are passing through zero modules, passing through one module, passing through two modules, and passing through three modules, respectively; curves “4” and “5” represent two different convolution operations, namely 3 × 3 transpose convolution and 3 × 3 slide convolution. In the horizontal direction (represents by *X_ij_*, *i* = 1, 2, 3 represents the resolution; *j* = 1, 2, 3 represents the information fusion stage), the high-resolution convolution information is expressed in stages as follows:

(2) $$\;X_{10}=x_{0}\;\;X_{11}=\left\{X_{10}+x_{0,1}\right\}\;\;X_{12}=\left\{X_{10}+X_{11}+x_{0,1,2}\right\}\;\;X_{13}=\left\{X_{10}+X_{11}+X_{12}+x_{0,1,2,3}\right\}\;$$

In the vertical direction (represented by *Y_ij_*, *i* = 1, 2, 3. *j* = 1, 2, 3), the three stages of information interworking of the three convolution channels are expressed as follows:

(3) $$Y_{13}=\left\{X_{12}+2X_{22}+4X_{32}\right\}\;\;Y_{23}=\left\{\left(1/2\right)X_{12}+X_{22}+X_{32}\right\}\;\;Y_{33}=\left\{\left(1/4\right)X_{12}+\left(1/2\right)X_{22}+X_{32}\right\}$$

The presentations of other convolution channels and information interworking stages can be inferred in the same way.

When the resolution changes from high resolution *X*_1_ to medium resolution *X*_2_, the 3 × 3 sliding convolution kernel was used to generate the feature map. During this process, the resolution was halved and the number of feature map channels was doubled. Conversely, when the resolution changed from medium resolution *X*_2_ to high resolution *X*_1_, the 3 × 3 transpose convolution kernel was used to generate the feature map. During this process, the resolution was doubled and the number of feature map channels was halved. 

**Figure 3:**
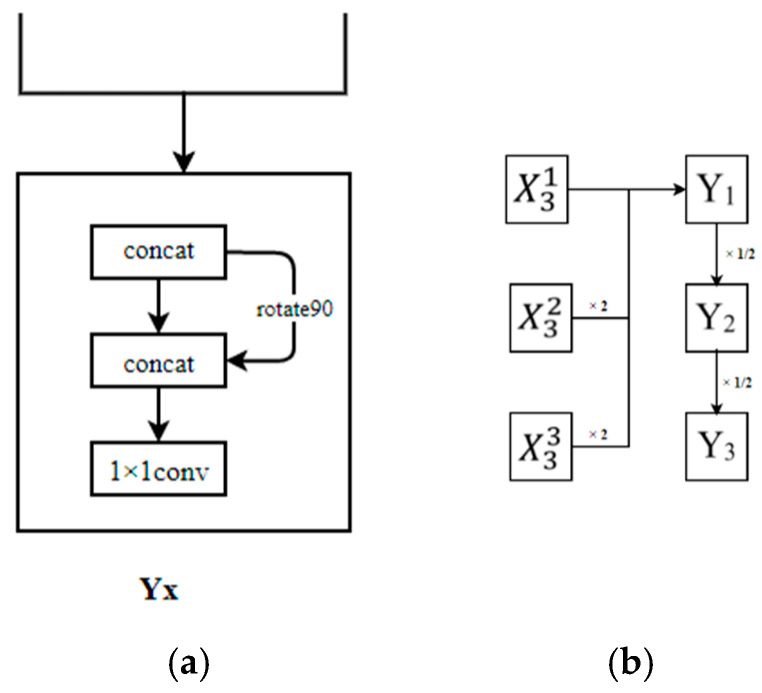

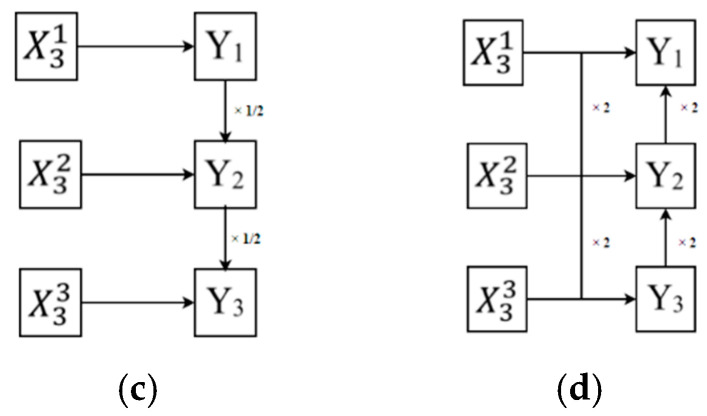
Network output schemes: (**a**) *Y_x_* module structure; (**b**) pyramid output mode; (**c**) advanced pyramid output mode; (**d**) high-resolution output mode.

In order to maximize utilization of the multiresolution convolution information in the network structure and produce the output of multiscale information on the premise of increasing only a few parameters and at a small amount of computational overhead, three multiscale output modes were designed, as shown in Figure 3. In the three output modes, three resolutions were used for data processing. Bilinear interpolation was used for upsampling from low to high resolution, average pooling was used for downsampling from high to low resolution and a 1 × 1 convolution kernel was used for backward transmission at the same resolution. The output module *Y_x_* (*x* = 1, 2, 3, etc.) is shown in Figure 3a. The input feature image was flipped 90 degrees and then operated by the concat function, and then a 1 × 1 convolution kernel was used for information interworking. $X_{3}^{i}$ (*i* = 1, 2, 3) represents the final result of information fusion at different resolutions.

In Figure 3b, the multiscale outputs are expressed as:

(4) $$\;\;Y_{1}=\left\{X_{3}^{1}+2X_{3}^{2}+4X_{3}^{3}\right\}\;\;Y_{2}=\left\{\left(1/2\right)Y_{1}\right\}\;\;Y_{3}=\left\{\left(1/2\right)Y_{2}\right\}$$

In Figure 3c, the multiscale outputs are expressed as:

(5) $$Y_{1}=\left\{X_{3}^{1}\right\}\;\;Y_{2}=\left\{X_{3}^{2}+\left(1/2\right)Y_{1}\right\}\;\;\;Y_{3}=\left\{X_{3}^{3}+\left(1/2\right)Y_{2}\right\}\;$$

In Figure 3d, the multiscale outputs are expressed as:

(6) $$Y_{3}=\left\{X_{3}^{3}\right\}\;\;Y_{2}=\left\{X_{3}^{2}+2X_{3}^{3}+2Y_{3}\right\}\;\;Y_{1}=\left\{X_{3}^{1}+2X_{3}^{2}+2Y_{2}\right\}\;$$

## 4. Experimental Results

### 4.1. Experimental Setup

#### 4.1.1. Dataset

The dataset used in this study mainly involved seven static scenes. The dataset included 5304 training images and 1769 test images with the size of 1280 pixels × 720 pixels. The training images contained 22,234 samples of the human body and 13,634 samples of the human head; the test images contained 7843 samples of the human body and 4513 samples of the human head. Unless otherwise specified, the dataset mentioned in this paper refers to this particular dataset.

#### 4.1.2. Evaluation Metrics

The *mAP* (mean average precision) is the most widely used performance indicator in the field of target detection. In this paper, precision *P*, recall *R*, and *mAP* were jointly used to compare and evaluate the detection performance of the proposed model. The values of *P* and *R* were respectively calculated by Equations (7) and (8):

(7) $$P=\frac{TP}{\left(TP+FP\right)}\;\;$$

(8) $$R=\frac{TP}{\left(TP+FN\right)}\;$$

where *TP* represents the true positive; *FP* represents the false positive; *FN* represents the false negative.

In order to obtain *P* and *R* values, it is necessary to calculate IoU so as to determine whether a result is correct. The threshold used in this study was set to 0.5; therefore, if IoU > 0.5, a sample was considered as *TP*; otherwise, it was considered as *FP*. For *P* and *R,* the confidence was obtained according to classification, and it was divided into 18 thresholds {0.1, 0.15, ..., 0.95}. As for *mAP*, the attention was mainly paid to AP50 (IoU0.5) and *mAP* (the mean value of *AP* under 10 IoU thresholds {0.50, 0.55, ..., 0.95}).

#### 4.1.3. Model Training

In this study, the most advantageous training parameters were determined by the analysis; the anchor length-width ratio of the anchor box was set to [0.3, 0.4, 0.5, 1.0]. Different training parameters were used for different algorithm networks. After passing the test, the SGD + COS learning and adjustment mode was used and multiscale training and verification mode was adopted. The parameter training scale was set to [(1280, 720), (1120, 630), (960, 540), (800, 450)] and the validation scale was set to [(1280, 720), (1120, 630), (960, 540)]. The input scales of training and verification were set according to the length–width ratio.

#### 4.1.4. Infer Metric

In this study, the reasoning performance of the network was tested using the Nsight Compute tool based on the MMdetecion framework. The Nsight Compute, which is an analysis tool used to test and optimize the performance of CUDA applications, can collect, view, and analyze data in the command lines. The parameters related to the network reasoning process, including the time consumption of each item and the proportion of time, are presented in Table 1.

The primary concern of this study was the reasoning performance of the designed model. Table 1 shows only part of the results and the parameter named CUDA memcpy HtoD indicates the time spent on copying image data from the host to the device. Omitting the calculation of the network reasoning time, the reasoning ability on a single image is expressed as Equation (9):

(9) $$Y=\frac{T_{C}}{P_{C}}-T_{C}\;$$

where $T_{C}$ represents the time spent on copying data of an item (CUDA memcpy HtoD); $P_{C}$ represents the percentage of data copying time in the total used time; *Y* represents the reasoning time consumed by a single image on the reasoning device, i.e., reasoning performance.

### 4.2. Ablation Study

#### 4.2.1. Model

Due to various reasons, in real-world applications, the volume of training data is usually limited. To address this limitation, a common practice is to perform basic weight training on other datasets first and then to retrain the trained model using the training data of the current project. A weight training dataset was designed for the proposed model, and it contained the following data:

(1) Classification weights trained on the ImageNet [27] dataset;
(2) Detection weights trained on the PHF COCO (person and head from MSCOCO) dataset [28] consisting of person and head data extracted from the MSCOCO (2014) dataset;
(3) Detection weights trained on 81 categories of the MSCOCO (2014) dataset.

**Figure 4:**
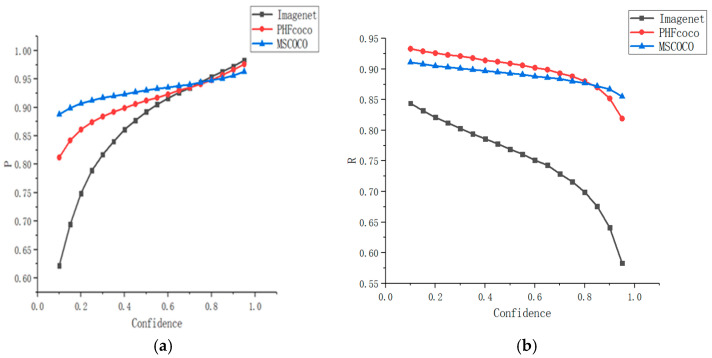
(**a**) Detection accuracy obtained on different datasets at IoU threshold of 0.5; (**b**) recall obtained on different datasets at the IoU threshold of 0.5.

Based on the basic weight training, three training schemes were designed. The IoU threshold was set to 0.5 and AP50 (AP—average precision) and AR50 (AR—average recall) were used as the evaluation criteria. The network detection result corresponding to AP50 is shown in Figure 4a, where the abscissa indicates the confidence interval and the ordinate indicates the percentage of accurate predictions. The network detection result corresponding to AR50 is shown in Figure 4b, where the abscissa indicates the confidence interval and the ordinate indicates the recall percentage. The importance of data to the network generalization was clearly verified by the results presented in Figure 4 and the sequence is as follows: ImageNet → PHFCOCO → MSCOCO. The accuracy improved significantly in the low-confidence section and dropped slightly in the high-confidence zone. The model trained on the MSCOCO dataset achieved a great improvement in recall (above 90%). In terms of the overall trend, the curve tended to be stable after pretraining and the prediction became more accurate, which was mainly due to the rich features of the MSCOCO dataset. The generalization ability of the proposed model was improved essentially by the implementation of migration learning based on the pretrained model; thus, the detection performance of the proposed model was greatly improved.

#### 4.2.2. Input Data

**Figure 5:**
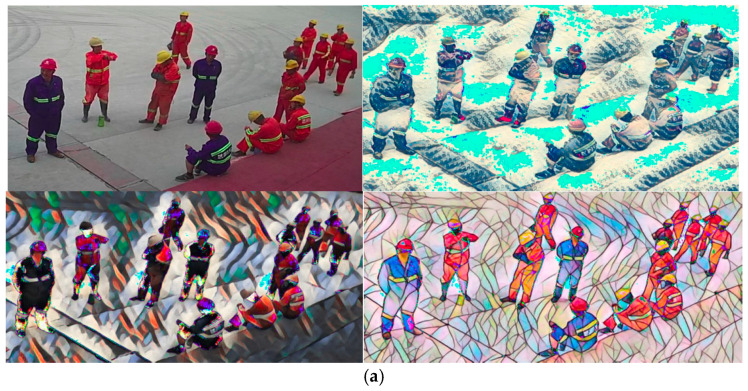

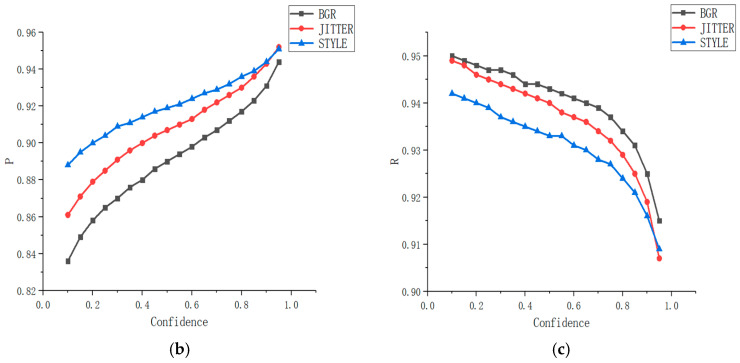
(**a**) The effect of performing augmentation transformation on datasets; (**b**) detection accuracy curves of the three schemes; (**c**) recall curves of the three schemes.

In the dataset used in this study, most of the targets were persons wearing maroon clothes and yellow helmets. Considering that the proposed model can be sensitive to the color jitter and that the dataset is relatively small in scale, the data augmentation strategy was adopted. Two strategies based on data analysis were designed: the JITTER strategy based on color dithering and contrast transformation of images and the STYLE strategy based on the offline style conversion of images. Considering the influence of data scenes, 10 different styles of augmentations were used randomly for scenes in the dataset during training. Several typical conversion effects are shown in Figure 5a. 

In this section, three experimental schemes are presented. The first scheme uses a BRG image as input data of the network. The second scheme is similar to the first scheme, except that it also accepts the data obtained by implementing the JITTER strategy on an image such as the input. The third scheme is similar to the second scheme, except that it also accepts the data obtained by implementing the STYLE strategy on an image as the input. On the premise that the accuracy could reach the detection standard, the latter two schemes achieved significantly higher detection accuracy compared to the first scheme. The detection performances of the three schemes are presented in Figure 5b,c.

#### 4.2.3. Convolution Module

**Figure 6:**
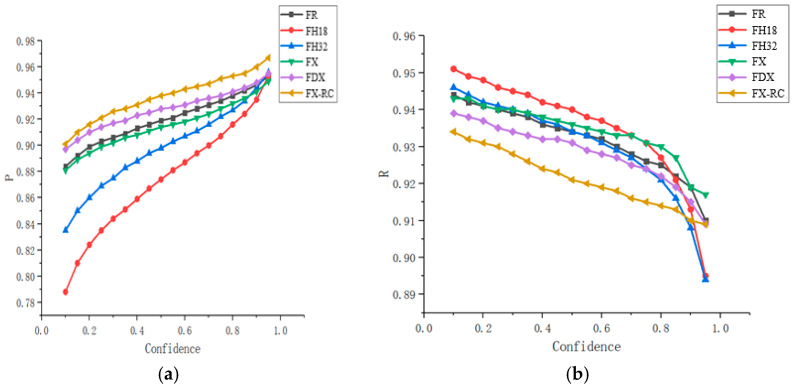
(**a**) Accuracy curves of the six experimental schemes; (**b**) recall curves of the six experimental schemes.

From the Inception V1-4 [29,30,31,32] and ResNet architectures to the Xception and ResNeXt architectures, researchers have been continually exploring appropriate width and depth values of a network, studying the fusion of information from the bottom and top network layers, and increasing the utilization of the underlying information in the network. The main goal has been to maximize the utilization and fusion of the convolution information at the lowest possible computational cost by processing the post-convolution information. In this section, the detection results of several basic network schemes using different convolution methods, including the ResNet101, HRNetv2p_w18, HRNetv2p_w32, ResNeXt101, Dconv_c3-c5_ResNeXt101, and ResNeXt-RC, were compared. On the premise of meeting the application requirements of the project, the evaluation criteria of the experimental results favored the highest possible detection effect while ensuring a recall rate over 90%. The results are shown in Figure 6a,b, where F stands for the Faster RCNN, R stands for the ResNet101, H18 stands for the HRNetv2p_w18, X stands for the ResNeXt101, DX stands for the Dconv_c3-c5_ResNeXt101, and FX-RC stands for the Faster RCNN network structure based on the ResNeXt-RC underlying network. The results showed that when the confidence threshold was in the range of [0.1, 0.8], the recall was over 90% in all cases. The detection accuracy curves are displayed in Figure 6a, where it can be seen that when the confidence threshold was in the range of [0.7, 0.95], the detection accuracy was always over 90%, and the scheme based on the ResNeXt-RC achieved the highest accuracy of 96.67%. These experimental results verified that the utilization of the underlying information and the design of the convolution module were successful. It should be noted that the larger network depth was beneficial in most cases, but in certain cases, its advantages cannot be demonstrated in specific tasks. Therefore, it is highly necessary to design an efficient and simple network for specific datasets and special scenarios.

#### 4.2.4. Detection Effect and Reasoning Performance

The main objective of this study was to improve the detection performance of the network while maintaining high reasoning performance. Much work has been done on the optimization and improvement of network depth and width and information exchange. Considering that different tasks have different requirements for performance and effect, nine schemes, including all the schemes mentioned in the previous section, were selected to perform network training and testing and different models comparison.

**Figure 7:**
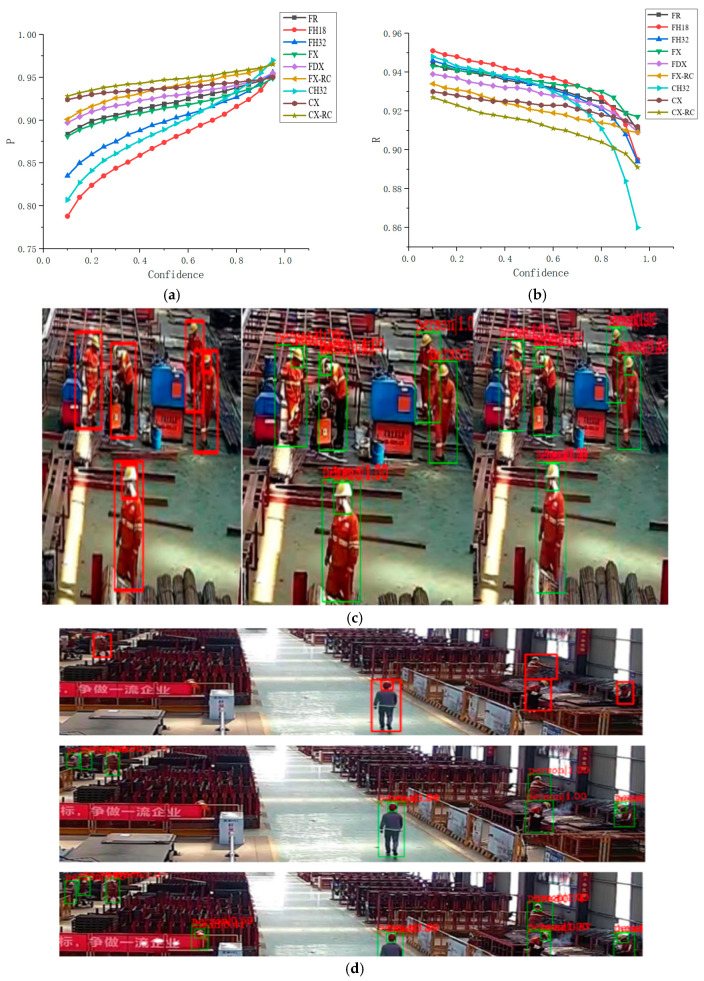
(**a**) Detection accuracy of the nine schemes obtained using the faster and cascade structures; (**b**) recall figures of the nine schemes; (**c**,**d**) detection results of two typical scenes.

These include ResNet101, HRNetv2p_w18, HRNetv2p_w32, ResNeXt101, Dconv_c3-c5_ResNeXt101, ResNeXt-RC based on the Faster RCNN structure and HRNetv2p_w32, ResNeXt101, and ResNeXt-RC based on the Cascade RCNN structure. The experimental reasoning platform included a NVidia GeForce GTX1080Ti 11G graphics card. The *P* and *R* curves plotted according to the test data are shown in Figure 7a,b, where the meaning of each curve was the same as in the previous figures, C stands for the Cascade RCNN. With regard to the detection accuracy, the network based on the cascade structure achieved an overall accuracy that was 2.5% higher than that achieved by the network based on the faster structure and the prediction stability of the former was also higher than that of the latter. The prediction accuracy of the network based on the faster structure fluctuated in the range of approximately 8%, while the accuracy fluctuation range of the network based on the cascade structure was only 3%. This was because the feature extraction operation was performed thrice during the detection process in the network based on the cascade structure. Based on the recall curves, the network based on the cascade structure achieved the absolute advantage in the detection accuracy on the premise that its performance in recall could meet the requirement of the task. The proposed network based on the ResNeXt-RC modular structure could also achieve high accuracy and its detection performance is shown in Figure 7c,d, where the red frames denote the annotation boxes of the dataset and the green frames are the detection box added to the experiment. In Figure 7c,d, from left to right and from top to bottom, the images denote the data annotation map, the detection result map of the network based on the faster-RCNN structure, and the detection result map of the network based on the cascade RCNN. The detection results showed that the network based on the cascade RCNN structure had a salient advantage in detecting small targets, which was mainly because it had two extra cascaded detectors compared to the network based on the faster-RCNN structure. Compared to the ResNeXt101 network based on the cascade structure, the proposed IIHNet network could reduce the reasoning time by approximately 50% without making a compromise in detection performance. The reasoning performance is given in Table 2.

The nine schemes were put under the multiscale reasoning performance test, in which three scales, namely, 1280 × 720, 960 × 540, and 800 × 450, were used. The analysis and performance calculations were conducted by the Nsight Compute tool and the calculation results for evaluating the reasoning performance were obtained by averaging the calculation data of three rounds. As shown in Table 2, the IIHNet network achieved significant improvement in reasoning performance when it was used on the dataset; it achieved the highest accuracy for the scale of 1280 × 720 and this accuracy was 20% higher than the second-highest accuracy achieved by the ResNeXt101 scheme. From the perspective of practical application, the IIHNet network based on the MMdetection framework could achieve an accuracy of 96.67% without any reasoning acceleration, which corresponded to the reasoning performance of 12 frames per second.

Table 2 shows the reasoning performance results of the nine schemes for different scales. The pretraining of each of the schemes was performed on the COCO dataset. In Table 2, “Input size” represents the size of the input image, and A, B, and C correspond to the scales of 1280 × 720, 960 × 540, and 800 × 450, respectively; “Infer” represents the reasoning performance and its unit is ms; *AP50* and *AP75* represent the average precisions at an IoU of 0.5 and 0.75, respectively.

## 5. Conclusions and Future Works

To meet the requirements of small-target detection, this paper proposed a new CNN-based network model named the IIHNet. The proposed model exhibited a considerable advantage in both the detection effect and reasoning performance. The results of the comparative experiment show that the IIHNet network improved the detection reasoning performance by 20–70% compared to the existing detection network models, such as the faster RCNN (Resnext101-DCONV, HRNet) and cascade RCNN (ResNeXt101), when tested on different datasets on the premise that its detection performance is on a par with or even exceeds that of those networks. 

There are three main reasons for the excellent performance of the proposed IIHNet network model and they are as follows: (1) the convolution module design based on information fusion; (2) information exchange among the convolution channels of different resolution and among different modules during the entire detection process; (3) multiscale network output mode.

In future works, the application scope of small-target detection will be expanded so to include satellite image detection, face detection, semantic segmentation, and other modern applications. The main aim is to develop smaller, faster, and stronger network strategies.

## Figures and Tables

**Table 1:** Parameters related to the network reasoning process.

Name	Time (ms)	Time (%)
CUDA memcpy HtoD	22.341	11.02
ROIAlignForward	24.959	12.32
Winnograd128128	56.824	28.04

**Table 2:** Analysis of inference performance of different scales based on nine schemes.

Metro	Backbone	Input Size	Infer	AP	AP50	AP75
		A	99.659	65.4	94.4	78.4
	ResNet101	B	65.962	63.5	93.5	73.5
		C	56.561	58.8	89.3	67.8
		A	79.884	64.1	94.6	77.4
	HRNetv2p_w18	B	55.735	63.6	93.8	76.2
		C	47.603	59.7	90.5	69.6
		A	98.435	64.3	93.9	77.1
Faster	HRNetv2p_w32	B	67.867	63.5	93.5	75.5
		C	59.478	58.9	88.8	68.4
		A	118.423	66.1	95.0	80.3
	ResNeXt101	B	80.673	64.2	93.5	76.1
		C	68.842	59.6	88.4	70.1
		A	143.38	65.5	94.1	78.8
	D_ResNeXt101	B	93.274	63.7	93.1	75.5
		C	77.849	59.2	88.4	69.3
		A	75.218	65.5	94.6	78.2
	ResNeXt-RC	B	54.026	63.4	93.5	73.2
		C	46.873	58.6	88.2	67.6
Cascade		A	135.19	65.4	93.8	79.1
	HRNetv2p_w32	B	104.731	64.5	93.5	77.7
		C	96.017	62.2	91.6	73.5
		A	147.471	66.3	94.0	79.6
	ResNeXt101	B	113.283	63.6	92.6	76.4
		C	100.857	61.7	90.8	73.6
		A	115.26	65.8	93.9	79.1
	ResNeXt-RC	B	96.037	64.6	93.5	77.2
		C	87.431	61.5	90.4	72.3

## Data Availability

Not applicable.
